# Maintenance of GLUT4 expression in smooth muscle prevents hypertension-induced changes in vascular reactivity

**DOI:** 10.14814/phy2.12299

**Published:** 2015-02-13

**Authors:** Kevin B Atkins, Yoshinori Seki, Jharna Saha, Felix Eichinger, Maureen J Charron, Frank C Brosius

**Affiliations:** 1 Department of Internal Medicine, University of Michigan Medical SchoolAnn Arbor, Michigan, USA; 2 Department of Biochemistry, Albert Einstein College of MedicineNew York City, New York, USA; 3 Obstetrics and Gynecology and Women's Health, Albert Einstein College of MedicineNew York City, New York, USA; 4 Medicine, Albert Einstein College of MedicineNew York City, New York, USA; 5 Department of Physiology, University of Michigan Medical SchoolAnn Arbor, Michigan, USA

**Keywords:** Contractility, COX-2, endothelial-dysfunction, GLUT4, IP-receptors, relaxation

## Abstract

Previous studies have shown that expression of GLUT4 is decreased in arterial smooth muscle of hypertensive rats and mice and that total body overexpression of GLUT4 in mice prevents enhanced arterial reactivity in hypertension. To demonstrate that the effect of GLUT4 overexpression on vascular responses is dependent on vascular smooth muscle GLUT4 rather than on some systemic effect we developed and tested smooth-muscle-specific GLUT4 transgenic mice (SMG4). When made hypertensive with angiotensin II, both wild-type and SMG4 mice exhibited similarly increased systolic blood pressure. Responsiveness to phenylephrine, serotonin, and prostaglandin F_2α_ was significantly increased in endothelium-intact aortic rings from hypertensive wild-type mice but not in aortae of SMG4 mice. Inhibition of Rho-kinase equally reduced serotonin-stimulated contractility in aortae of hypertensive wild-type and SMG4-mice. In addition, acetylcholine-stimulated relaxation was significantly decreased in aortic rings of hypertensive wild-type mice, but not in rings of SMG4 mice. Inhibition of either prostacylin receptors or cyclooxygenase-2 reduced relaxation in rings of hypertensive SMG4 mice. Inhibition of cyclooxygenase-2 had no effect on relaxation in rings of hypertensive wild-type mice. Cyclooxygenase-2 protein expression was decreased in hypertensive wild-type aortae but not in hypertensive SMG4 aortae compared to nonhypertensive controls. Our results demonstrate that smooth muscle expression of GLUT4 exerts a major effect on smooth muscle contractile responses and endothelium-dependent vasorelaxation and that normal expression of GLUT4 in vascular smooth muscle is required for appropriate smooth muscle and endothelial responses.

## Introduction

The insulin responsive glucose transporter, GLUT4, is expressed in vascular smooth muscle (VSM) (Brosius et al. 1992; Marcus et al. 1994; Banz et al. 1996; Bergandi et al. 2003). We have previously shown that GLUT4 participates in constitutive, noninsulin-dependent glucose uptake in arterial VSM (Atkins et al. 2001; Park et al. 2005). This unusual property distinguishes VSM from other tissues that express GLUT4 as in those tissues GLUT4 largely resides in intracellular vesicles until translocated to the plasma membrane in response to insulin or other physiologic stimuli (Bryant et al. 2002). In addition, we have reported that GLUT4 expression is decreased in large arteries of hypertensive rats and mice (Atkins et al. 2001, 2005; Park et al. 2005) and that, similar to arteries from hypertensive animals, arterial reactivity in arteries from GLUT4 knockout mice is increased compared to vessels from wild-type (WT) animals (Park et al. 2005). Using whole body GLUT4 overexpressing mice, we demonstrated that the preserved expression of GLUT4 in DOCA-salt hypertension prevented the enhanced response to serotonin (5-HT) observed in aortae of hypertensive WT mice (Atkins et al. 2007). These latter results left open the possibility that systemic metabolic changes resulting from whole body GLUT4 overexpression, and not GLUT4 overexpression specifically in vascular smooth muscle, were responsible for the salutory effects observed in vascular reactivity.

To test whether vascular smooth muscle GLUT4 expression prevented the vascular contractile abnormalities in hypertensive vessels, we generated and studied a mouse model that overexpressed GLUT4 only in smooth muscle. Here, we report that maintenance of vascular smooth muscle GLUT4 expression prevents development of enhanced vasoconstrictive responses in hypertension. Moreover, persistently normal vascular smooth muscle GLUT4 expression dramatically ameliorated endothelial dysfunction observed in hypertension. These results confirm and extend our previous findings suggesting that loss of vascular smooth muscle GLUT4 expression leads to abnormal vasoreactivty in hypertension and implicate complex mechanisms by which maintenance of vascular smooth muscle GLUT4 expression can normalize defects in endothelium-dependent vasorelaxation.

## Materials and Methods

### Animal models

**Figure 1:**
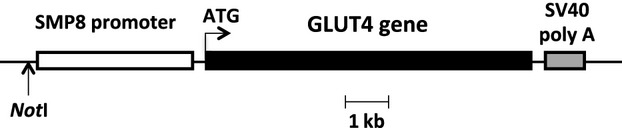
Schematic of the smooth muscle GLUT4 (SMG4) transgene. The 3.6 kb mouse SMP8 promoter was cloned upstream of the mouse GLUT4 coding sequences followed by a 850-bp SV40 poly A tail.

The smooth-muscle-specific GLUT4 transgene (SMG4) was constructed using the 3.6 kb mouse smooth muscle α-actin promoter (SMP8; Wang et al. 1997) ligated upstream of 7.5 kb of the mouse GLUT4 gene (Tsao et al. 1996). An 850 bp fragment containing the SV40 poly A site was ligated to the 3′ end of the GLUT4 gene for transcript stability (Fig.1). The transgene fragment was purified following restriction with *Not*I and electrophoresis on a 1.0% agarose gel for excision. Standard pronuclear microinjection into FVB fertilized eggs and implantation into foster females was completed at the Transgenic Mouse Facility of the Albert Einstein College of Medicine. GLUT4 transgenic animals were identified by PCR amplification of tail DNA using primers 5′ GCT ACT GCT GAC TCT CAA CAT T 3′ and 5′ GGA CAA ACC ACA ACT AGA ATG C 3′, and 5′ TCC TCA AAG ATG CTC ATT AG 3′ and 5′ GTA ACT CAC TCA TGC AAA GT 3′ which amplify a 600-base pair or a 400-base pair product, respectively, from SMG4, and WT mice. The SMG4 transgene was backcrossed onto the C57BL/6J background for >10 generations prior to initiating the experiments detailed below. Transgene expression was confirmed in SMG4 mice using total RNA extracted from various tissues using the TRIZOL Reagent (Life Technologies) according to the manufacturer's instructions. Approximately 8 μg of total RNA was loaded onto a 1.2% formaldehyde-agarose gel, transferred to a Hybond-N nylon membrane (Amersham, Piscataway, NJ), then hybridized overnight to a random primed ^32^P-labeled poly A probe under high-stringency conditions (50% formamide, 42°C). The filter was washed at 42°C in 0.2x SSC, 0.1% SDS solution and subjected to phosphoimage analysis.

### Induction of hypertension

Adult male mice (25–30 g) were anesthetized with isofluorane and a 14-day osmotic mini-pump (Durect, Cupertino, CA) containing vehicle or AngII (Phoenix Pharmaceuticals, Burlingame, CA) to deliver at the rate of 1.4 mg/kg/d was placed subcutaneously in the mid-scapular region. Systolic blood pressure in conscious mice was measured by the tail-cuff method (CODA, Kent Scientific, Torrington, CT) after 2 weeks of AngII infusion. Mice were then anesthetized with pentobarbital (50 mg/kg i.p.) and both muscles of the diaphragms were cut. The thoracic aorta (from aortic arch to diaphragm) from each animal was removed, carefully cleaned and either used for reactivity or snap frozen in liquid nitrogen and stored at −80°C. The procedures used in this study were approved by the University of Michigan Committee on the Use and Care of Animals and the Albert Einstein College of Medicine Institutional Animal Care and Use Committee. The University of Michigan Unit for Laboratory Animal Medicine provided veterinary care for the animals reported in this study. Both the University of Michigan and Albert Einstein College of Medicine are accredited by the American Association of Laboratory Animal Care. The animal care and use programs conform to the standards in “The Guide for the Care and Use of Laboratory Animals,” Department of Health, Education, and Welfare Publication No. 86-23.

### Immunoblotting

Frozen vascular samples were pulverized by bead-homgenization (TissueLyser, Qiagen, Germantown, MD), suspended in the presence of sodium dodecyl sulfate (SDS)-polyacrylamide gel electrophoresis (PAGE) loading buffer (125 mmol/l Tris-HCl, pH 6.8, 4% SDS, 20% glycerol, 100 mmol/l PMSF, 10 mg/mL aprotinin, 1 mg/mL leupeptin, 1 mg/mL pepstatin A), and sonicated, as previously reported (Atkins et al. 2005, 2007; Park et al. 2005). Protein concentration was determined (Pierce BCA, Thermo, Rockford, IL) and lysates (50 μg) were run on 7.5 or 10% SDS-PAGE and immunoblotted with antibodies for GLUT4 (MW∼55 kD – Abcam, Cambridge, MA), pERM (MW∼90 kD), pMYPT (MW∼130 kD – Thr850), and COX-2 (MW∼72 kD – Santa Cruz Biotechnology, Santa Cruz, CA), and MYPT (MW∼130 kD – BD Biosciences, San Jose, CA). All blots were within the linear range and, with the exception of those for pMYPT (normalized to total MYPT), were normalized to SM α-actin (MW∼41 kD) or α-actinin (MW∼100 kD – Sigma, St Louis, MO) the expression of which we have found are unaffected by hypertension (Armoni et al. 2014). Immunoblots for GLUT4 exhibit multiple bands due to the highly glycosylated nature of this protein (Brosius et al. 1992; Katz et al. 1995; Atkins et al. 2001, 2005, 2007; Park et al. 2005).

### Vascular reactivity experiments

These experiments were performed as previously detailed (Marchesi et al. 2013). Briefly, mouse aortae were cleaned and cut into 3-mm length rings. The aortic rings were mounted in a myograph system (Danish Myo Technology A/S, Aarhus, Denmark). Vessels were bathed with warmed (37°C), aerated (95% O_2_/5% CO_2_) physiological salt solution (PSS, mmol/L: NaCl 130, KCl 4.7, KHPO_4_ 1.18, MgSO_4_ 1.17, CaCl_2_ 1.6, NaHCO_3_ 14.9, dextrose 5.5, CaNa_2_ EDTA 0.03). Rings were set at 700 mg passive tension and equilibrated for 1 h, washing every 20 min. Prior to performing concentration–response curves, vessels were contracted with isotonic PSS containing 60 mmol/L KCl in which an equimolar quantity of KCl was substituted for NaCl (KPSS) for 10 min. After washing out the 60 mmol/L KPSS, the vessels were contracted with 100 mmol/L KPSS, allowed to plateau, which was followed by washout. Cumulative concentrations of phenylephrine (PE), serotonin (5-HT), and prostaglandin F_2_α (PGF_2_α) (Sigma Chemical, St. Louis, MO) were added to the bath to establish a concentration–response contraction curve. Contractions were expressed as a percent of the 100 mmol/L KPSS contraction. Rings were contracted with a concentration of PE equal to that which stimulated 80% of the maximum force generated in the cumulative curve. After allowing the contraction to plateau, cumulative concentrations of acetylcholine (Ach) were added to establish a concentration–response relaxation curve. Rings similarly contracted to PE were also treated with cumulative concentrations of sodium nitroprusside (SNP). In both instances, Ach and SNP (Sigma Chemical, St. Louis, MO), the force is expressed as a percent of that achieved with 80% PE. In all cases where the effect of inhibition (Y-27632, CAY10404, CAY10441; Cayman Chemical, Ann Arbor, MI) was tested separate rings from the same aorta were treated with or without the inhibitor. Inhibitor was added 30 min. prior to agonist treatment.

### Statistical evaluation

Data were expressed as means ± SEM. Unpaired two-tailed Student's *t*-tests were used to compare results from two groups. For the vascular reactivity experiments, agonist EC50 values were calculated with a nonlinear regression analysis with the algorithm [effect = maximum response/1 +  (EC50/agonist concentration)] using GraphPad Prism (San Diego, CA). For all other comparisons ANOVA with Bonferroni post-hoc analysis was performed. Differences were considered to be statistically significant when *P *<* *0.05.

## Results

**Figure 2:**
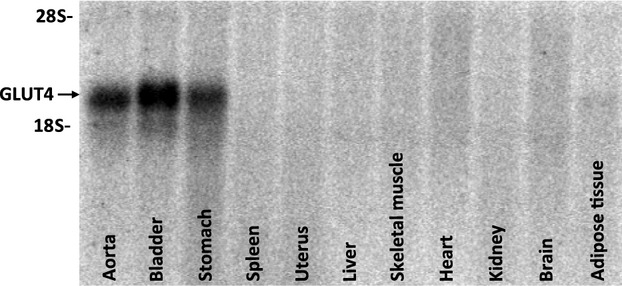
Tissue distribution of GLUT4 transgene mRNA expression. Northern blotting with a Poly A-specific probe showed that transgene expression was limited to smooth muscle and was absent from traditionally high GLUT4 expressing tissues such as heart, skeletal muscle, and adipose tissue (*n* = 5).

**Figure 3:**
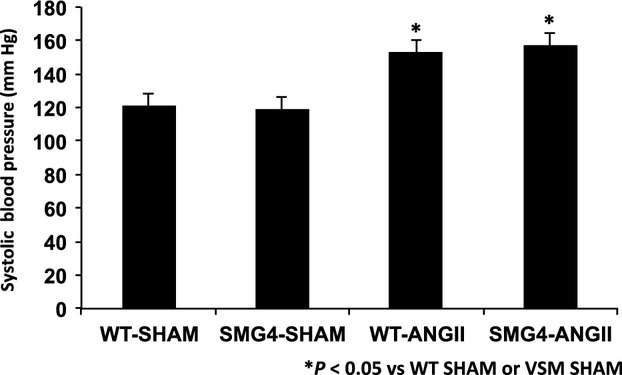
Effect of Angiotensin II (AngII) treatment on systolic blood pressure in wild-type (WT) and GLUT4 transgenic (SMG4) mice. Blood pressure was taken by tail-cuff at the end of 2 weeks. These data represent greater than ≥3 separate trials with *n* = 5/group.

SMG4 mice were generated as described in the Materials and Methods (Fig.1). Using RNA from a large panel of tissues, only aorta, bladder, and stomach expressed the SMG4 transgene (Fig.2). In contrast to the whole body GLUT4-transgenic mice we studied previously (Atkins et al. 2007), the SMG4 transgene was not expressed in major GLUT4-expressing, insulin responsive tissues (adipose tissue, heart, skeletal muscle; Fig.2). Systolic blood pressure significantly increased following 2 weeks of AngII treatment in both WT and SMG4 mice compared to the respective Sham-treated mice. There was no difference in systolic blood pressure between the two AngII-treated groups (Fig.3).

**Figure 4:**
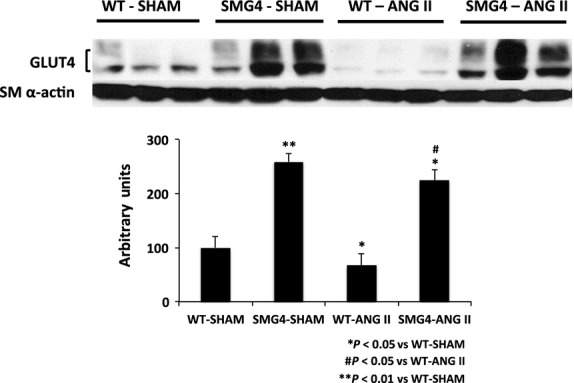
The GLUT4 expression in aortae of Sham- or AngII-treated wild-type or SMG4 mice. The representative GLUT4 immunoblot of aortic lysates (50 μg) was performed after 2 weeks of treatment and combined data (bar graphs) were derived from ≥3 separate trials (*n* = 5/group). Order of lanes of scanned radiographs may have been rearranged to reflect convention of figure and does not alter the information contained therein.

As previously reported in several rodent models of hypertension (Atkins et al. 2001, 2005, 2007; Park et al. 2005), immunoblots demonstrated that GLUT4 expression was significantly decreased in aortae of WT-Ang II-treated animals (Fig.4). GLUT4 levels were significantly higher in aortae of SMG4-Sham mice than in aortae of WT-Sham mice, and were not significantly decreased in aortae of SMG4-Ang II mice compared to SMG4-Sham mice.

**Figure 5:**
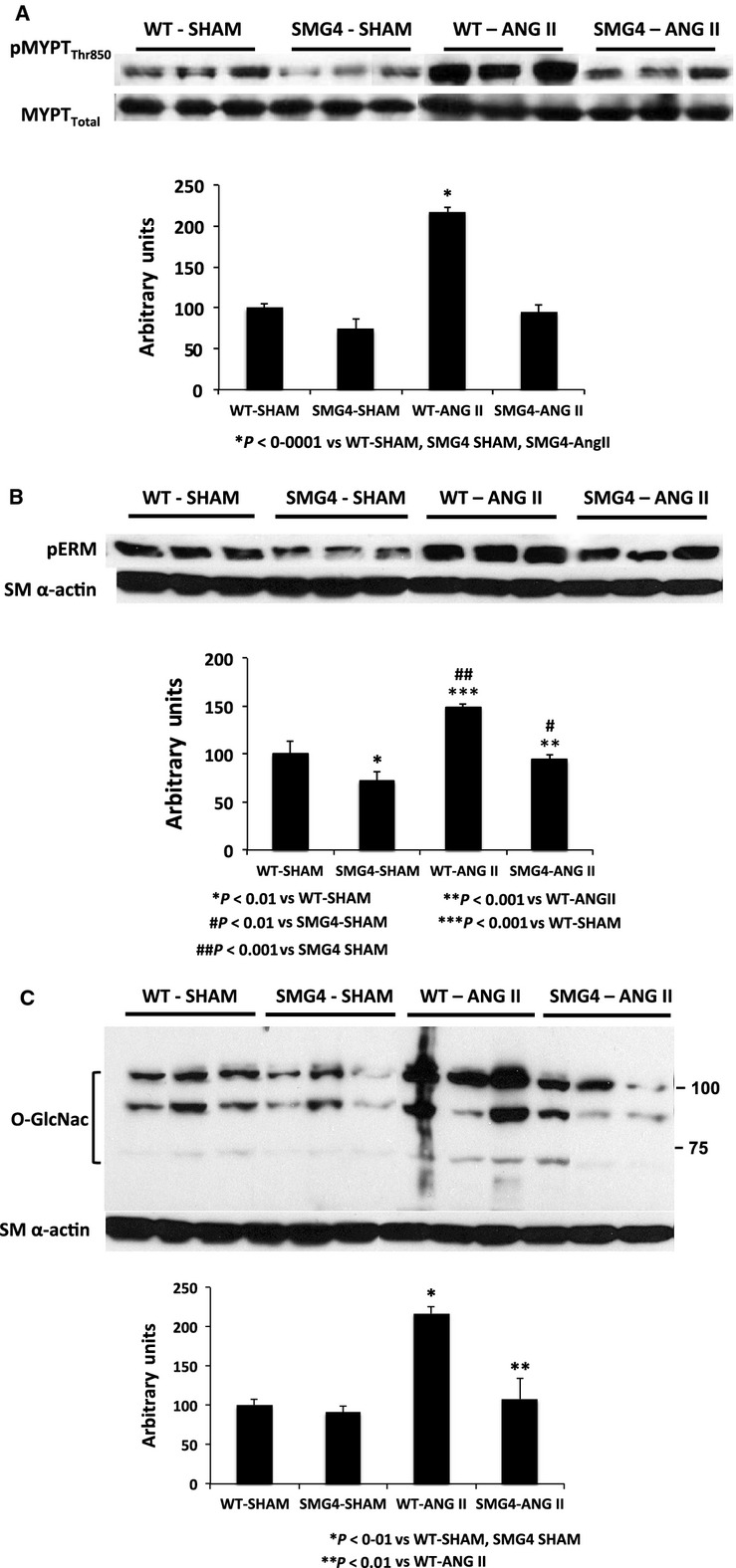
Expression of targets of Rho-kinase phosphorylation. Myosin targeting subunit Thr_850_ (A), Ezrin, Radixin, Moesin (ERM; (B) and O-GlcNac-labeled proteins (C). Immunoblots of aortic lysates (50 μg) were performed after 2 weeks of AngII treatment and combined data (bar graphs) were derived from ≥3 separate trials (*n* = 5/group). Order of lanes of scanned radiographs may have been rearranged to reflect convention of figure and does not alter the information contained therein.

In whole body GLUT4-transgenic mice we previously observed differential aortic expression of phosphorylated myosin phosphatase targeting subunit (pMYPT) compared to aortae of WT mice (Atkins et al. 2007). Here, phosphorylation of MYPT was not significantly different in aortae of SMG4 compared to WT mice, however, pMYPT was significantly increased in aortae of WT-AngII-treated mice compared to WT-Sham, SMG4-Sham, and SMG4 AngII mice (Fig.5A). Similar results were observed for phosphorylation of ERM (Ezrin, Radixin, Moesin; Fig.5B) as well as for levels of O-GlcNAcylation of aorta proteins (Fig.5C). However, pERM levels in aortae of SMG4-Sham mice were significantly lower than those in WT-Sham mice (Fig.5B).

**Figure 6:**
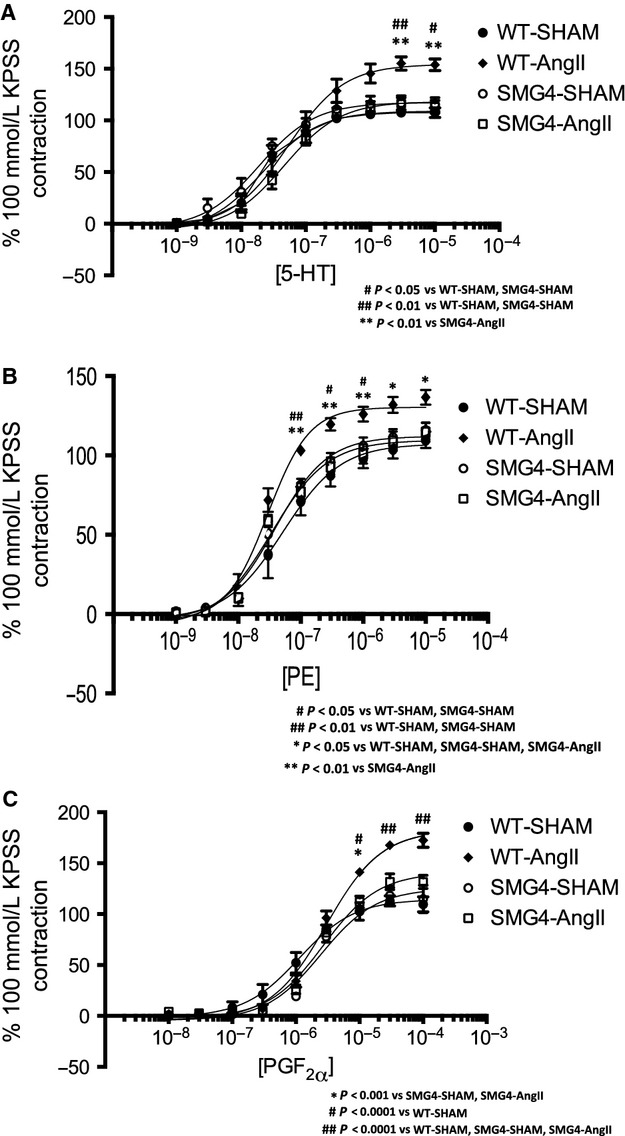
Reactivity to serotonin (5-HT; A), phenylephrine (PE; B), and prostagandin F_2α_ (PGF_2α_; C) by endothelium-intact aortic rings from Sham- or AngII-treated wild-type or SMG4 mice after 2 weeks of treatment. Concentration–response curves are expressed as a percentage of the contraction elicited by 100 mmol/L KPSS. Data were derived from ≥3 separate trials (*n* = 5/group).

Next we assessed maximal vascular contractile response. The maximal contractile response to three agonists (5-HT, PE and PGF_2_α) was significantly greater in aortae of WT-AngII mice compared to all other groups (Fig.6A–C). Furthermore, there was an increase in potency (EC_50_) with 5-HT and PGF_2_α but not with PE in aortae from WT-AngII compared to SMG4-AngII mice (Table1). Thus, AngII increased the maximal contractile response to all three agonists in WT but not SMG4 aortae. AngII also increased the contractile potency of two of the agonists (5-HT and PGF_2_α) in WT but not in SMG4 aortae (Table1).

**Table 1:** Potency of 5-HT, PE, and PGF2_α_ in thoracic aorta of WT or SMG4 mice with or without angiotensin II (AngII)

	−logEC_50_		
	5-HT	PE	PGF_2α_
WT-SHAM	7.661 ± 0.065^*^	7.279 ± 0.113	5.949 ± 0.087^#^
WT-Angll	7.273 ± 0.102	7.534 ± 0.057	5.524 ± 0.044^*^^*^
SMG4-SHAM	7.721 ± 0.111^*^	7.411 ± 0.073	5.607 ± 0.096
SMG4-Angll	7.336 ± 0.071^*^	7.473 ± 0.077	5.567 ± 0.057^*^^*^

Values are mean ± SE; *n* ≥ 15. ^*^P <* *0.001 versus WT-AngII; ^*^^*^P <* *0.01 versus WT-SHAM; #*P *<* *0.05 versus SMG4-SHAM; by one-way ANOVA with Bonferroni multiple comparisons post hoc test.

**Figure 7:**
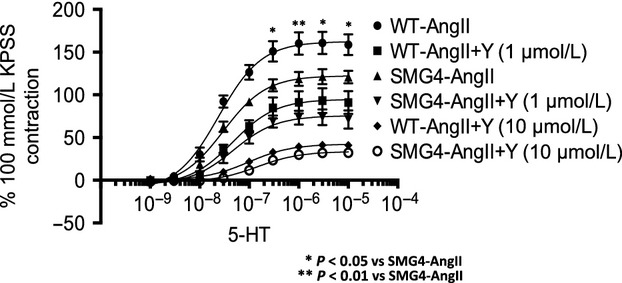
Effect of inhibition of Rho-kinase on 5-HT stimulated contractility of endothelium-intact aortic rings of wild-type and SMG4 mice after 2 weeks of AngII treatment. Concentration–response curves are expressed as a percentage of the contraction elicited by 100 mmol/L KPSS. Data were derived from ≥3 separate trials (*n* = 5/group).

Given that expression of markers of enhanced Rho-kinase activity (Fig.5A–C.) were unchanged in aortae of SMG4-AngII mice, we explored whether modulation of ROCK accounted for effects of GLUT4 expression on vascular contractility. Using 5-HT, because of the demonstrated difference in both maximum response and sensitivity with AngII, we compared responsiveness in aortae from WT-AngII and SMG4-AngII mice at two concentrations of the Rho-kinase inhibitor, Y-27632 (Fig.7; Table2). The extent of inhibition (percentage of control *E*_max_) elicited by both concentrations of Y-27632 was similar in WT-AngII and SMG4-AngII (41.6% vs. 38.3% and 73.9% vs. 72.5% at 1 μmol/L and 10 μmol/L, respectively) aortae (Fig.7; Table2).

**Table 2:** Potency of 5-HT with or without inhibition of Rho-kinase by Y-27632 in thoracic aorta of WT or SMG4 mice treated with angiotensin II (AngII)

	*E*_max_5-HT_	
	WT-AngII	SMG4-AngII
Control	162.3 ± 8.27	122.4 ± 3.38
1 µmol/L Y-27632	94.78 ± 5.17	75.56 ± 3.73
10 µmol/L Y-27632	42.32 ± 1.42	33.66 ± 1.71

Values are mean ± SE; *n* ≥ 15.

**Figure 8:**
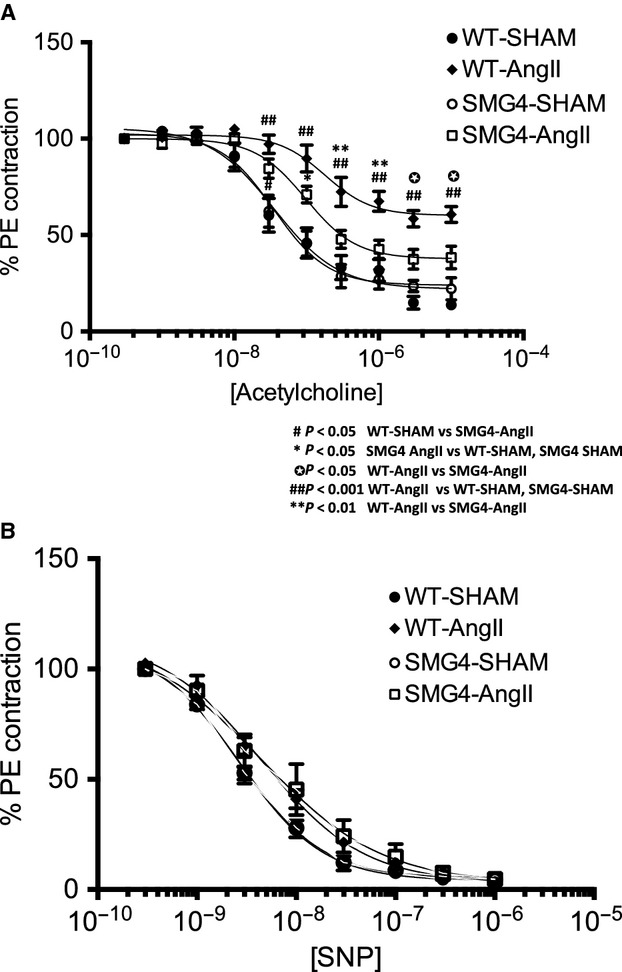
Acetylcholine- (A) or sodium nitroprusside-stimulated (B) relaxation in aortic rings of Sham- or AngII-treated wild-type or SMG4 after 2 weeks of treatment. Force is expressed as a percent of that achieved with 80% PE (see methods). Data were derived from ≥3 separate trials (*n* = 5/group).

Maximum responsiveness and potency to acetylcholine-stimulated relaxation was unchanged in SMG4-Sham compared to that in WT-Sham aortae (Fig.8A; Table3). Acetylcholine-stimulated maximum relaxation and sensitivity were significantly decreased in WT-AngII aortae (Fig.8A; Table3). In contrast, maximum relaxation and sensitivity to acetylcholine in SMG4-AngII aortae was not statistically different. Maximum responsiveness, but not sensitivity, to acetylcholine was significantly increased in WT-AngII compared to SMG4-AngII aortae (Fig.8A; Table3). Sodium nitroprusside stimulated relaxation was similar in aortae from all groups (Fig8B).

**Table 3:** Potency of Ach in thoracic aorta of WT or SMG4 mice treated with or without angiotensin II (AngII)

	−logEC_50Ach_
WT-SHAM	7.459 ± 0.119
WT-Angll	6.702 ± 0.168^*^
SMG4-SHAM	7.446 ± 0.102
SMG4-Angll	7.006 ± 0.102

Values are mean ± SE; *n* ≥ 15. ^*^P <* *0.01 versus WT-Sham, SMG4-SHAM by one-way ANOVA with Bonferroni multiple comparisons post hoc test.

**Figure 9:**
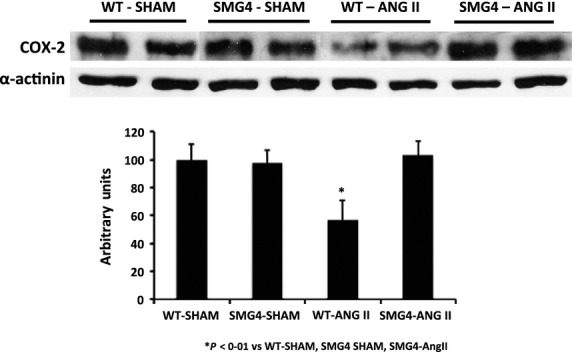
The COX-2 expression in aortae of Sham- or AngII-treated wild-type or SMG4 mice. The representative GLUT4 immunoblot of aortic lysates (50 μg) was performed after 2 weeks of treatment and combined data (bar graphs) were derived from ≥3 separate trials (*n* = 5/group). Order of lanes of scanned radiographs may have been rearranged to reflect convention of figure and does not alter the information contained therein.

**Figure 10:**
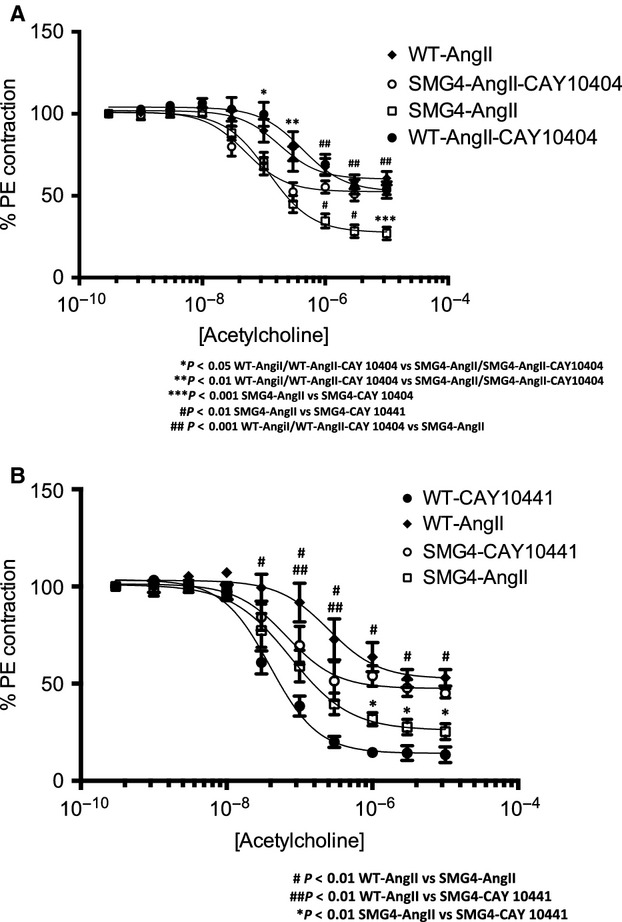
Effect of inhibition of COX-2 (A) or prostacyclin receptors (IP; B) on acetylcholine-stimulated relaxation in aortic rings of wild-type and SMG4 mice after 2 weeks of AngII treatment. Force is expressed as a percent of that achieved with 80% PE (see methods). Data were derived from ≥3 separate trials (*n* = 5/group).

Given evidence suggesting a pathogenic role for COX-2 in endothelial dysfunction (Wang et al. 1997; Seko et al. 2003; Cannon and Cannon 2012; Virdis et al. 2013; Wong et al. 2013) we investigated the possibility that the effect of maintenance of GLUT4 expression on endothelial function might be via inhibition of COX-2. Interestingly, we found that COX-2 expression was significantly reduced in aortae from WT-AngII but not SMG4-AngII mice (Fig.9). We therefore explored the functional role of COX-2 in the differential responsiveness to acetylcholine noted above. Treatment with the specific COX-2 inhibitor, CAY10404 (1 μmol/L), had no effect on the relaxation observed in WT-AngII aortae (Fig.10A). In contrast, CAY10404 evoked a significant reduction in acetylcholine-stimulated relaxation in SMG4-AngII aortae (Fig.10A). This result indicated a vasodilatory product of COX-2 action, such as prostacyclin, might be responsible for the difference in relaxation observed between WT-AngII and SMG4-AngII aortae. We therefore evaluated the effect of an inhibitor of the prostacyclin activated IP-receptor, CAY10441 (5 μmol/L), on Ach-stimulated relaxation of aortae. Indeed IP-receptor signaling blockade inhibited the Ach-stimulated relaxation of SMG4-AngII aortae (Fig.10B). The final acetylcholine-stimulated (*E*_max_) relaxation was not significantly different in aortae from SMG4-AngII mice treated either with CAY10404 (COX-2 inhibitor) or CAY10441 (IP-receptor blocker) compared to that observed in aortae of WT-AngII mice (Fig.10A and B). Interestingly, relaxation of WT-AngII aortae was significantly increased by CAY10441 treatment (Fig.10B).

## Discussion

We have previously demonstrated that arterial smooth muscle expression of GLUT4 is reduced in several rodent models of hypertension (Atkins et al. 2001, 2005, 2007; Park et al. 2005). These models also show enhanced arterial sensitivity to agonists such as 5-HT and ergonovine. Whether there is a causal connection between reduced GLUT4 expression and functional vascular abnormalities seen in hypertension remained to be tested. We previously demonstrated that increased total force generation, as well as enhanced sensitivity to agonist stimulation, occurred in arteries of normotensive GLUT4 null mice (Park et al. 2005). In addition, transgenic overexpression of GLUT4 in all GLUT4-expressing tissues prevented the hypertension-enhanced vascular reactivity seen in several rodent models of hypertension, despite failing to reduce blood pressure (Atkins et al. 2007). While these studies suggested a direct effect of vascular smooth muscle GLUT4 on vascular reactivity, they did not rule out the possibility that metabolic changes due to systemic deletion or overexpression of GLUT4 accounted for these observations. To address this question, we generated a smooth-muscle-specific GLUT4 transgenic mouse to determine the direct effect of arterial smooth muscle GLUT4 expression on vascular reactivity.

As observed with transgenic mice overexpressing GLUT4 in all GLUT4-expressing tissues, smooth-muscle-specific GLUT4 overexpression had no effect on the development of hypertension. Conversely, GLUT4 overexpression in smooth muscle prevented all contractile abnormalities seen in the hypertensive vessels thereby confirming a critical role for normal or elevated GLUT4 in vascular smooth muscle in hypertension. It is important to emphasize that the effect of increased GLUT4 expression on vasoreactivity is only observed in the context of hypertension. There was no apparent contractile phenotype in the normotensive SMG4 mice compared to WT, despite the significant differences in GLUT4 expression. However, whereas GLUT4 expression decreased in aortae of hypertensive compared aortae of normotensive wild-type mice, there was not a significant decrease in GLUT4 between the two conditions in aortae of SMG4 mice. Significant changes in vascular responses were only observed between aortae of normotensive and hypertensive wild-type mice (i.e., those aortae in which GLUT4 expression had significantly decreased). Thus, the prevention of enhanced contractility observed in hypertensive SMG4 mice was likely due to maintaining GLUT4 expression during hypertension and not an effect of GLUT4 overexpression.

The current results and those reported previously demonstrated that, at least in conduit arteries, there is an uncoupling of increased reactivity and hypertension. As we previously reported (Atkins et al. 2007) there was increased phosphorylation of MYPT, the targeting subunit of myosin phosphatase, in aortae of hypertensive WT mice. This increase in phospho-MYPT was prevented by specifically overexpressing GLUT4 in the smooth muscle of these arteries. This targeting subunit, is a substrate of Rho-kinase (ROCK), and ROCK activation is associated with increased vasoreactivity in hypertensive rodents (Weber and Webb 2001; Seko et al. 2003; Lee et al. 2004; Hilgers et al. 2007). While it is believed that the Thr850 residue of MYPT is targeted by ROCK (Ito et al. 2004; Grassie et al. 2011), in this study we demonstrated that two other markers of ROCK activation were also increased in aortae of hypertensive mice. The phosphorylation of ezrin, radixin, and moesin (ERM) and acylation with O-linked B-N-acetylglucosamine (O-GlcNac) of serine and threonine residues of cellular proteins were increased in aortae of WT-hypertensive mice. While there was a significant increase in phospho-ERM in aortae of SMG4-AngII mice compared to that in SMG4 mice, the former phosphorylation levels were similar to those in aortae of WT nonhypertensive mice. As previously demonstrated in DOCA-salt rats (Lima et al. 2009), O-GlcNAcylation was increased in angiotensin II hypertensive WT mice and was prevented by smooth muscle GLUT4 overexpression in hypertensive SMG4 mice. These observations suggest that maintaining the expression of smooth muscle GLUT4 prevented or significantly limited the activation of ROCK in hypertensive aortae.

Given the potentially causal role of ROCK activation in the contractile abnormalities of hypertensive WT mice and the apparently reduced ROCK activation in hypertensive SMG4 mice, we investigated the possibility that ROCK might be an intermediary in the mechanism of GLUT4 effects on contractility. Our findings suggest that the differences in observed contractility to 5-HT between WT-AngII and SMG4-AngII aortae were not explained by changes in ROCK activity as the response to 5-HT at each concentration of Y-27632 was the same in aortae of WT-AngII and SMG4-AngII mice. Thus, the mechanism of reduced contractility observed in aortae of hypertensive SMG4 mice was not dependent upon decreased ROCK activation.

While ROCK activation was not essential, the prevention of increased responsiveness to contractile agonists in the aortae of hypertensive SMG4 mice nonetheless may have been due to preserved myosin light chain phosphatase activity. Phosphorylation of MYPT leads to reduced myosin phosphatase activity (Hirano et al. 2003, 2004), and increased reactivity by increased calcium sensitization resulting from inhibition of dephosphorylation of myosin light chain by myosin phosphatase (Hirano et al. 2003, 2004; Lee et al. 2004). By whatever mechanism, maintenance of GLUT4 was associated with suppression of hypertension-induced increased expression of pMYPT (Atkins et al. 2007). Moreover, reduced GLUT4 levels and increased pMYPT led to an enhanced response to contractile agonists (Park et al. 2005; Atkins et al. 2007). This association is supported by our previous finding that GLUT4 ablation results in increased MYPT phosphorylation and increased contractility in normotensive GLUT4 knockout mice (Park et al. 2005). These latter observations demonstrated functional effects of GLUT4 on vascular responsiveness even in the absence of underlying cardiovascular pathology.

Interestingly, this study found that maintenance of GLUT4 expression in vascular smooth muscle prevented the development of endothelial dysfunction (reduced endothelium-dependent relaxation to acetylcholine) typically exhibited by aortae of WT-hypertensive mice. The somewhat surprising observation that a protein expressed exclusively in smooth muscle can regulate endothelial responsiveness has precedence in previously published results that demonstrated impaired endothelium-dependent relaxation in tissue-specific knockout of the vascular smooth muscle PPARγ (Marchesi et al. 2013).

Our data suggest that the mechanism by which GLUT4 modulated endothelial function in hypertensive SMG4 aortae was via COX-2 activation. Inhibition of COX-2, as well as inhibition of IP-receptors, in aortae of hypertensive SMG4 mice reduced endothelium-dependent relaxation to the level found in aortae from hypertensive WT mice. These results suggest that decreased relaxation in aortae of hypertensive WT mice, due to decreased expression of GLUT4, was mediated by reduced COX-2-dependent prostacyclin production. In support of this hypothesis, immunoblots demonstrated a reduction in COX-2 expression in aortae of hypertensive WT mice compared to that in aortae of hypertensive SMG4 mice. Vascular-specific COX-2 deletion has been shown to result in endothelial dysfunction (Yu et al. 2012). Moreover, prostacyclin biosynthesis was suppressed by vascular smooth-muscle-specific deletion of COX-2 (Yu et al. 2012). Our finding that relaxation in aortae of hypertensive WT mice was enhanced by IP-receptor blockade was opposite what we anticipated. The concentration of the IP-receptor (blocker) used in these experiments also inhibited U46619-mediated vasoconstriction (not shown) suggesting dual blockade of IP and TP (thromboxane A_2_) receptors by this inhibitor. Thus, elevated thromboxane A_2_ activity may be present in the aortae of hypertensive WT mice which has been shown to be involved in decreased acetylcholine-dependent relaxation (Wong and Vanhoutte 2010). It is also possible that there was an excess production of prostacyclin in the hypertensive WT mice aortae as it has been demonstrated that in some vascular pathologic conditions excess prostacyclin production leads to activation of TP receptors (Wong and Vanhoutte 2010), although we did not observe the endothelium-dependent contractions associated with this phenomena.

In summary, we have shown that maintenance of GLUT4 expression in aortae of mice, which is decreased with hypertension, was sufficient to block hypertension-induced increased arterial contractility and decreased arterial relaxation. It is probable that the effect of GLUT4 on contractility was associated was mediated by the phosphorylation status of MYPT. However, the most interesting finding was that maintenance of vascular smooth muscle GLUT4 expression exerted profound effects on endothelial function. The mechanism for this effect appeared to involve COX-2 activity and prostacyclin signaling through IP-receptors. The mechanism whereby changes in GLUT4 expression modulated MYPT phosphorylation, COX-2 activation and downstream production of prostacyclin remains to be elucidated.

## Limitations

Our previous results (Atkins et al. 2007) and those reported herein demonstrate that maintaining GLUT4 expression in aortae of hypertensive mice prevents increased responses to contractile agonists. Our data demonstrate these effects in a conduit vessel, but leaves open the question of what is the response in resistance vessels. Our results, both previously (Atkins et al. 2007) and here, demonstrate that there is no significant blood pressure difference between hypertensive wild-type and transgenic mice. This suggests that there is no difference in contractile agonist-stimulated responses between resistance vessels of hypertensive wild-type and transgenic mice given the role of resistance vessels in peripheral resistance and hypertension (Lund-Johansen 1983; Mulvany 2005). Confirmation of this supposition would, however, be of interest in a follow-up study.

The consideration of the lack of blood pressure differences begs the question of the sensitivity of the tail-cuff measurement we employed. Measurement of rodent blood pressure using the VPR method employed herein has been demonstrated to show good agreement with those obtained by telemetry (Feng et al. 2008). Whether we may have missed a small but significant difference in the elevated level of blood pressure that could explain differences in response to agonist stimulation is unlikely given that the actual extent of increase (from normotensive to hypertensive) in blood pressure observed in the two genotypes was similar if not actually larger in the transgenic mice.

We do not know the mechanism by which GLUT4 gene expression affects vascular responses in hypertensive aortae. We have demonstrated that COX-2 is involved in this mechanism, but do not know how GLUT4 expression modulates COX-2 activity. While GLUT4 expression also modulates Rho-kinase activity, this effect does not seem to be associated with the GLUT4-induced changes in vascular responses that we have found.

## Conflict of Interest

None declared.
